# Feature-Based Laser Scan Matching and Its Application for Indoor Mapping

**DOI:** 10.3390/s16081265

**Published:** 2016-08-10

**Authors:** Jiayuan Li, Ruofei Zhong, Qingwu Hu, Mingyao Ai

**Affiliations:** 1 Beijing Advanced Innovation Center for Imaging Technology, Capital Normal University, Beijing 100048, China; zrf@cnu.edu.cn; 2 School of Remote Sensing and Information Engineering, Wuhan University, Wuhan 430079, China; ljy_whu_2012@whu.edu.cn (J.L.); aimingyao@whu.edu.cn (M.A.); 3 State Key Laboratory of Information Engineering, Surveying, Mapping and Remote Sensing, Wuhan University, Wuhan 430079, China

**Keywords:** scan matching, point and line features, indoor mapping, relative pose estimation, lq-norm

## Abstract

Scan matching, an approach to recover the relative position and orientation of two laser scans, is a very important technique for indoor positioning and indoor modeling. The iterative closest point (ICP) algorithm and its variants are the most well-known techniques for such a problem. However, ICP algorithms rely highly on the initial guess of the relative transformation, which will reduce its power for practical applications. In this paper, an initial-free 2D laser scan matching method based on point and line features is proposed. We carefully design a framework for the detection of point and line feature correspondences. First, distinct feature points are detected based on an extended 1D SIFT, and line features are extracted via a modified Split-and-Merge algorithm. In this stage, we also give an effective strategy for discarding unreliable features. The point and line features are then described by a distance histogram; the pairs achieving best matching scores are accepted as potential correct correspondences. The histogram cluster technique is adapted to filter outliers and provide an accurate initial value of the rigid transformation. We also proposed a new relative pose estimation method that is robust to outliers. We use the lq-norm (0 < *q* < 1) metric in this approach, in contrast to classic optimization methods whose cost function is based on the l_2_-norm of residuals. Extensive experiments on real data demonstrate that the proposed method is almost as accurate as ICPs and is initial free. We also show that our scan matching method can be integrated into a simultaneous localization and mapping (SLAM) system for indoor mapping.

## 1. Introduction

The mapping of indoor environments or underground spaces has become increasingly important in recent years. However, the lack of availability of GPS signals inside buildings and underground spaces makes indoor modeling and mapping a challenging task. Recently, numerous works have appeared to solve this issue. The most popular solution may be the simultaneous localization and mapping (SLAM) technique, which is widely applied in robotics. Compared with vision-based SLAM systems, laser-based ones can provide more accurate indoor maps and models.

The core of laser-based SLAM, scan matching, is a technique to recover the relative position and orientation of two laser scans. It estimates a rigid transformation to project one laser scan so that the projected laser scan aligns with the other one. The ICP algorithm [1,2] and its variants (to name a few [3,4]) are used pervasively in laser scan matching. However, the performance of ICPs is unstable. When the good initial guess of the transformation is unavailable, the ICPs may not converge to a correct solution. Thus, ICP-based SLAM systems usually fuse additional highly expensive sensor data for guaranteeing robustness, such as those from inertial measurement unit (IMU) and odometry. This will reduce the power of these methods for practical applications because of the very expensive hardware systems.

In vision-based SLAM systems, visual features (such as SIFT [5], SURF [6], ORB [7], and BRISK [8]) are adapted for relative pose recovery. Keyframe image matching typically consists of four major stages: keypoint detection, keypoint description, keypoint matching, and transformation estimation. In the first stage, salient and stable interest points are extracted. The keypoint is then described based on its photometric neighborhoods, such as local gradients. The third stage calculates the distances between the descriptor vectors to recognize reliable correspondences (inliers). Finally, the transformation matrix is computed based on RANSAC [9]. This pipeline has no assumption regarding the initial guess of the transformation.

Currently, 3D indoor mapping is drawing increasing attention. It can directly produce dense point clouds and does not rely on the prior of planar floors. However, 2D indoor mapping is still very important. The 2D indoor maps can be used for emergencies, evacuation and localization. For example, Google indoor maps are produced by a 2D indoor mapping system called Cartographer. To obtain 3D indoor models, another two 2D lasers can be integrated. Moreover, 2D lasers are much cheaper than 3D ones, and the processing of 2D laser scans is much more efficient than that of 3D scans.

In this paper, we carefully design a feature-based framework for 2D laser scan matching. Like keyframe image matching, there are also four basic stages in our framework. First, distinct feature points are detected based on an extended 1D SIFT, and line features are extracted via a modified Split-and-Merge [10,11] algorithm. The point and line features are then described by a distance histogram; the pairs that achieve the best matching scores are accepted as potential correct correspondences. Finally, we estimate the relative positions and orientations of two laser scans based on a robust cost function. Extensive experiments on real data demonstrate that the proposed method is almost as accurate as ICPs and is initial free. We also show that our scan matching method can be integrated into a SLAM system for indoor mapping and indoor modeling. The contributions of our work are summarized as follows:

(1) We propose a new initial-free 2D laser scan matching method by combining point and line features, which is a very effective technique for indoor mapping and modeling.
(2) We carefully design a framework for the detection of point and line feature correspondences from laser scan pairs. We also give an effective strategy to discard unreliable features. Thus, our detected feature correspondences are distinct, reliable, and invariant to rotation changes.
(3) We propose a new relative pose estimation method that is robust to outliers. We use the lq-norm (0 < *q* < 1) metric in this approach, in contrast to classic optimization methods whose cost function is based on the l_2_-norm of residuals. Unlike the conventional RANSAC-based [9] strategy, strategy, there is no gross error detection stage in our pose estimation algorithm. In addition, our pose estimation algorithm is more robust to noise than the RANSAC-based one.
(4) We make an honest attempt to present our work to a level of detail allowing readers to re-implement the method.

## 2. Related Work

Many works on 2D laser scan matching have been presented, so we do not give here a comprehensive study. We will review only a few most relevant and recent works, including the most well-known ICP families, feature-based methods, and other techniques.

The idea of the ICP algorithm [1,2] is intuitive and simple: it alternates between finding closest point correspondences under an initial transformation and fitting the transformation with the correspondences until convergence. The classical ICP algorithm is to minimize a function of point-to-point distance. Lu and Milios [12] proposed two ICP variants for scan registration. The first one alternates between translation estimation and rotation computation. Given a fixed rotation, the translation is optimized by least squares. The translation is then fixed, and the rotation is searched by the global-section method [13]. The second is an iterative dual correspondence (IDC) method that adapts two types of correspondences, i.e., Euclidean distance and range angular distance. Minguez et al. [14] presented a new metric distance for ICP, called MbICP, which considers the configuration space of the sensor and combines the rotation and translation errors of the sensor. As shown in their paper, MbICP improves the correspondence building stage and the convergence rate of the classical ICP. Censi [4] describes a point-to-line metric-based ICP (PLICP). In contrast to other point-to-line methods [2], PLICP develops a closed-form solution for the planar case, just like 2D laser scans. PLICP also improves the convergence of classical ICP from linear to quadratic. ICPs can achieve very high accuracy in scan matching. However, as mentioned earlier, their high dependence on a good initial guess will reduce their power for practical applications.

Diosi and Kleeman [15,16] developed a point-to-point matching method called polar scan matching (PSM). It directly matches the range measurement of two laser scans in the native polar coordinate system. PSM uses the matching bearing rule to associate the range measurements of the target scan with the reference scan and minimizes a weighted range residual cost function. This method is much faster than ICPs because it avoids searching for correspondences. As noted in [17], PSM can also improve the ability to converge to an optimal solution from a laser range initial guess.

Montesano et al. [18] give a probabilistic formulation of scan registration called probabilistic scan matching (Probabilistic SM). Like classical ICP, it also consists of two stages, i.e., correspondence location and transformation estimation. In the first stage, the uncertainty in both relative transformation and range measurement is modeled based on Gaussian distributions. In addition, Probabilistic SM allows these correspondences to be found by probability integration over all possible associations between the range measurements of the two scans. The probabilistic modeling of uncertainty is more suitable for real sensor data compared with pure geometrical approaches.

Another probabilistic-based method, called correlative scan matching (CSM) [19], uses cross-correlation to register two laser scans, which achieves real-time performance with little loss of accuracy. CSM maximizes the probability of having observed range measurements. That is, it searches for an optimal rigid transformation that overlaps the two laser scans as much as possible. To avoid a local maximum, CSM searches over the entire parameter space of plausible rigid-body transformations. To detect the plausible region, some additional information should be provided, such as that from visual odometry, wheel odometry, or commanded motion.

These techniques also rely on a good initial guess or prior information. To eliminate the dependence on assumptions, feature-based scan matching methods have appeared. Ramos et al. [20] propose a novel feature-based approach based on conditional random fields (CRF). In their CRF-Matching, a joint estimation over all observations in a laser scan is performed, and shape information is considered to reject outliers. The CRF model could integrate multiple features, such as shape features and appearance features. The maximum posteriori of the rigid transformation is estimated by loopy belief propagation. The main limitations are the high computational complexity and the unreliability for partially overlapping laser scan pairs. Tipaldi and Arras [21,22] presented a method called FLIRT. In their work, they studied three types of laser feature detectors (range-based, normal-based, and curvature-based detectors) and two feature descriptors (linear local shape context descriptor and β-Grids descriptor. Based on the comprehensive analysis of these detectors and descriptors, they combine the best detector with the best descriptor to form FLIRT. They show that FLIRT can be adapted in laser SLAM systems and that impressive results can be achieved. However, their descriptor in FLIRT is very slow, which prevents it from being widely used in real SLAM applications because the number of scans in a SLAM process is usually huge. In addition, there is no outlier removal section in their literature.

Motivated by these techniques, we propose a real-time scan matching approach based on point and line features. Our method is almost as accurate as ICPs and is initial free. It can be easily adapted in a real indoor SLAM system, as shown in our experiments.

## 3. Scan Matching

We first define some notations for this task. The right subscription k, k ∊ Z^+^ is used to indicate the frame of laser scans. *S_k_*, *P_k_* and *L_k_* represent the laser scan measurements, point features, and line segment features of the frame *k*. Let *fp*_(*k,i*)_(*x*_(*k,i*)_,*y*_(*k,i*)_) be the *i*-th feature point in *P_k_* and *fj*_(*k,i*)_ be the *i*-th feature line in *L_k_*. Thus, our scan matching problem can be described as:

Problem: Given two consecutive 2D laser scans *S_k_*_-1_ and *S_k_*, recover the relative rigid transformation (relative position ***t****_k_*_-1_ and orientation ***r****_k_*_-1_) ***T****_k_*_-1_ = {***r****_k_*_-1_, ***t****_k_*_-1_} from these two laser scans based on point and line feature correspondences.

### 3.1. Feature Detection

**Figure 1:**
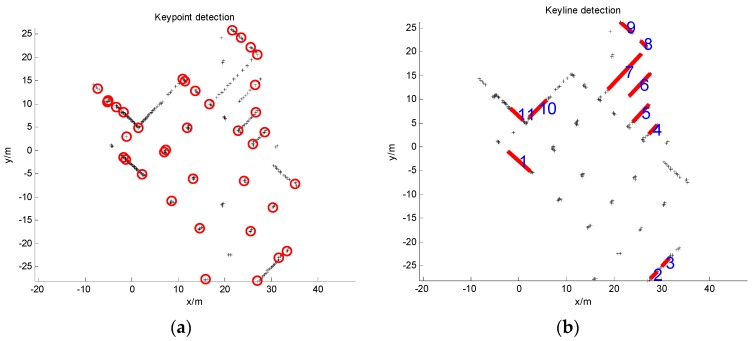
Point and line features detected from a laser scan. (**a**) feature point detection; (**b**) feature line detection.

*Keypoint detection*: The scale-invariant feature transform (SIFT) [5] is widely adapted in computer vision applications because of its robustness to image scale, rotation, illumination and viewpoint changes. In this stage, we propose an extended 1D SIFT keypoint detector so that it can be suitable for 1D range data (Figure 1a). We extract the keypoints in scale space only based on the raw laser range information.

The SIFT keypoint detector is based on the scale-space theory [23], which is a multiscale signal representation technique. The scale space uses a set of smoothed signals *Sig*(*x*, σ*_i_*) to represent the original signal *Sig*(*x*), where σ*_i_* is the size of the smoothing kernel, whose role is to suppress the fine scale structures. In our task, *Sig*(*x*) is the range information *S_k_*(*x*) of the *k*-th frame laser scan. The formulation of the scale-space construction is as follows:

(1) $$S_{k}(x,\sigma_{i})=K(x,\sigma_{i})*S_{k}(x)$$

where *K*(*x*, σ*_i_*) is a scale-space kernel. In the original SIFT, the Gaussian function is adopted because it is the only possible choice for continuous signals, as evidenced by Lindeberg [23]. However, laser range scans are 1D discrete signals. A discrete kernel, maintaining the properties of the Gaussian function, is chosen:

(2) $$K(x,\sigma_{i})=e^{-\sigma_{i}}I_{x}(\sigma_{i})$$

where *I_x_* stands for the modified Bessel function.

In SIFT, scale selection is performed to make detected feature points invariant to scale changes. However, the range data of different scans have the same scale. This means that the detected laser feature points are inherently invariant to scale. Thus, instead of extracting local extrema among three scale layers, we regard the local extrema of the Laplacian of the range signal at each scale layer *S_k_*(*x*, σ*_i_*) as feature keypoints. The Laplacian function is equivalent to the second derivative of *S_k_*(*x*,σ*_i_*) for one-dimensional signals. The keypoints *P_k_* are the local peaks of Equation (3):

(3) $$\nabla^{2}S_{k}(x,\sigma_{i})=S_{k}(x+1,\sigma_{i})+S_{k}(x-1,\sigma_{i})-2S_{k}(x,\sigma_{i})$$

**Figure 2:**
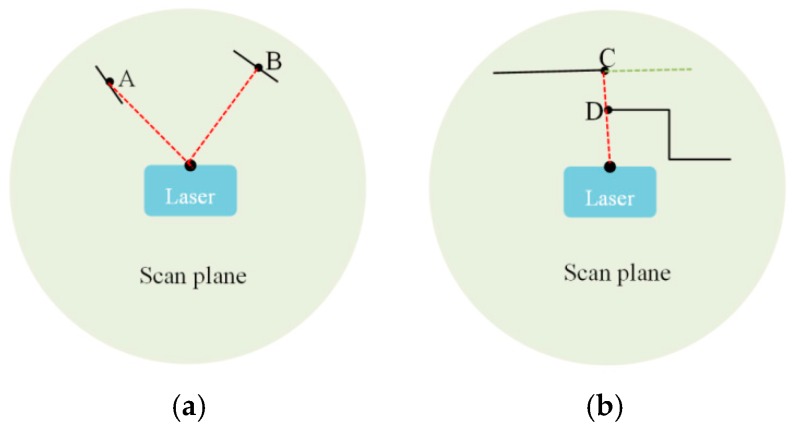
The black solid lines are the local surface patches of feature points; the red dotted lines are the laser beams; the green dotted lines represent the occluded region. (**a**) The direction of point A’s local surface patch is almost parallel to the laser beam, so the location accuracy of point A is very limited; (**b**) There is a gap between point C and point D, so point C may be occluded in the next scan. Point A and point C are both rejected as unreliable feature points in our approach.

For stability, we want to reject unreliable feature points whose local surfaces are roughly parallel to the laser beams (Figure 2a) or the points on the edges of occluded areas (Figure 2b). Point A is an unreliable feature point with low position accuracy because its local normal direction is almost perpendicular to the laser beam. Point C is also rejected because it may be occluded in the next laser scan. For example, if the laser of Figure 2b moves to the right side of the current position, point C will no longer exist. In detail, we calculate the local surface direction of each feature point and its distance to the neighboring points. If the difference between the local surface direction and laser beam is less than 10 degrees or one of the distances to the neighboring points is longer than 1 m, the feature point will be discarded.

*Keyline detection*: Line segments are a very important type of feature for indoor environments. The advantages of using line features are twofold: first, line features are more distinct for indoor scenes and are less sensitive to the noise in the laser range measurements; second, the orientation information can be easily extracted from 2D line correspondences, which can help us reject feature point outliers and provide a good initial guess for our transformation estimation stage. These advantages will be demonstrated in the following sections.

We detect keylines for each laser scan based on a slightly modified Split-and-Merge algorithm [10,11] (Figure 1b). First, consecutive laser points in laser scan *S_k_* are segmented into clusters, which, because of the points in the same cluster, tend to belong to the same object. The distance of two consecutive laser points is chosen as the segmentation criterion. The two points will be classified into the same cluster if the distance is smaller than a threshold. Clusters with too few points are regarded as isolated points and discarded. Then, for a cluster, we fit a line segment and detect the point with maximum distance to the line. If the maximum distance is smaller than a given threshold, we advance to process the next cluster. Otherwise, we split the cluster into two subclusters at the detected point. This process is recursive. When all possible lines are extracted, we calculate their slopes. Most 2D range finders are time-of-flight lidars, so the adjacent lines can be easily extracted. If two adjacent lines are almost parallel, we compute the distance between their middle points along normal direction. The two adjacent lines will be merged into one single line segment as long as the corresponding middle point distance is very small. Finally, the line segments with small length or few points are removed as unreliable keylines. The details are summarized in Algorithm 1.
**Algorithm 1.** Line Segment Extraction1 **input:** a laser scan $S_{k}$
2 **output:** line segments $L_{k}$
3 **begin**4  segment the laser scan $S_{k}$ and remove small    segments to form clusters $C_{k}$;5  **for** each cluster $c_{i}\in C_{k}$
**do**6   fit a line fl to $c_{i}$, compute the length $d_{l}$ of fl;7   remove line if its length is small;8   detect point p with maximum distance $d_{\max}$ to fl;9   **if**
$d_{\max}<\tau_{d}(\tau_{d}=0.1)$
**then**10    add the line segment fl to $L_{k}$;11   **else**12    split $c_{i}$ into two subclusters $sc_{i1}$, $sc_{i2}$ at p,     then, perform **Algorithm 1** for each subcluster;13   **end**14  **end**15  computer the slope $s_{fl_{\left(k,i\right)}}$ of each line segment in $L_{k}$,   find adjacent line pairs in $L_{k}$;16  **for** each pair $\left({fl}_{\left(k,i\right)}{fl}_{\left(k,j\right)}\right)$
**do**17   **if**
$\left|s_{fl_{\left(k,i\right)}}-s_{fl_{\left(k,j\right)}}|<\tau_{s}(\tau_{s}=3^{\circ }\right)$
**then**18    calculate their middle point distance in normal direction $d_{m}$;19    **if**
$d_{\max}<\tau_{d_{m}}(\tau_{d_{m}}=0.03)$
**then**20     merge $\left(fl_{\left(k,i\right)},fl_{\left(k,j\right)}\right)$ into a single line, update $L_{k}$;21    **end**22   **end**23  **end**24  **return** line segment set $L_{k}$;25 **end**

### 3.2. Feature Description

In this section, we use a distance histogram to describe point and line features. The distance histogram is different from the 2D gradient histogram because distance is invariant to rotation. Thus, our distance histogram descriptor is inherently invariant to rotation changes.

*Keypoint description*: For each feature point *fp*_(*k,i*)_ ∊ *P_k_*, we search its neighborhoods. If the distance between the searched point *p*_(*k,i*)_*S_k_* and feature point *fp*_(*k,i*)_ is less than the search radius *r*, the searched point *p*_(*k,i*)_ is considered to be a neighbor of the feature point *fp*_(*k,i*)_. These distances of the neighborhoods are then formed into an 8-bin histogram. The description of feature point *fp*_(*k,i*)_ is the normalized distance histogram.

**Figure 3:**
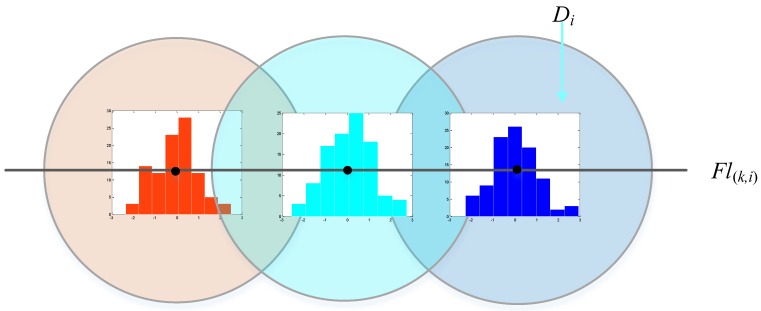
Illustration of constructing our line descriptor.

*Keyline description*: For each feature line *fl*_(*k,i*)_ ∊ *L_k_*, we find three points on the line (see Figure 3). These three points divide the feature line *fl*_(*k,i*)_ into four segments with equal length. Each point is then described by an 8-bin normalized distance histogram just like the feature point description. The description of feature line *fl*_(*k,i*)_ is a 24-bin normalized distance histogram that simply concatenates the three-point descriptor.

### 3.3. Feature Matching

**Figure 4:**
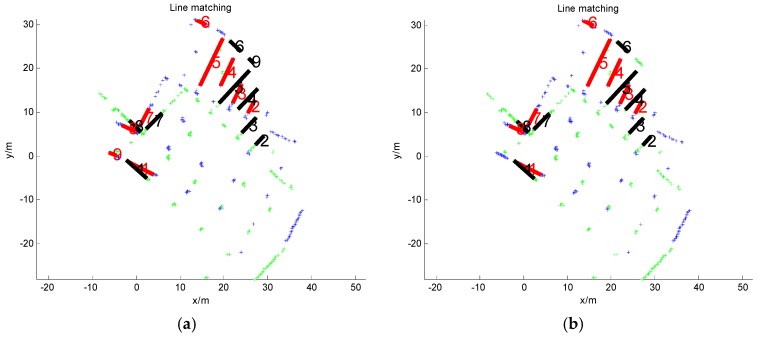
Feature line matching. (**a**) Line correspondences with outliers; (**b**) line matching result after outlier removal.

*Keyline matching*: For two consecutive laser scans *S_k_*_-1_ and *S_k_*, we detect their feature line segments *L_k_*_−1_ and *L_k_*. For each feature line *fl*_(*k*-1*,i*)_ ∊ *L_k_*_-1_, our goal is to search for its correspondence in *L_k_*. We first compute the matching score between *fl*_(*k*-1,*i*)_ and all lines in *L_k_*:

(4) $$score=∥dl(fl_{\left(k-1,i\right)})-dl(fl_{\left(k,j\right)})∥\;s.t.\;fl_{\left(k,j\right)}\in L_{k}$$

where dl(·) stands for the line descriptor; *score* is the matching score; and ∥·∥ represents the Euclidean distance. The feature line *fl*_(*k,j*)_ ∊ *L_k_* that achieves the best matching score will be accepted as the potential correspondence of *fl*_(*k*-1*,i*)_. We then check the lengths of *fl*_(*k*-1*,i*)_ and *fl*_(*k,j*)_. They are discarded as outliers if their length difference is very large because the line correspondence pair in laser scans usually has similar *S_k_*_-1_ lengths. Figure 4a shows a sample result. As seen, most feature lines are correctly matched. However, there are still some outliers, such as line correspondence 9. Because the *L_k_* relationship between laser scans and *S_k_* is rigid transformation, the angles or rotations between the correct 2D line correspondences are almost the same. Thus, we first cluster the angles of line correspondence via the histogram technique. The average angle of the largest cluster is regarded as the correct relative rotation between *S_k_*_-1_ and *S_k_*, formatted as matrix ${\bar{r}}_{k-1}$. The correspondences with angles that are inconsistent with ${\bar{r}}_{k-1}$ are rejected. We then rotate the line segments in and calculate the middle point distance between the remaining matches. The correspondences with middle point distances that are too different from others are also treated as outliers. The others are accepted as inliers (Figure 4b).

**Figure 5:**
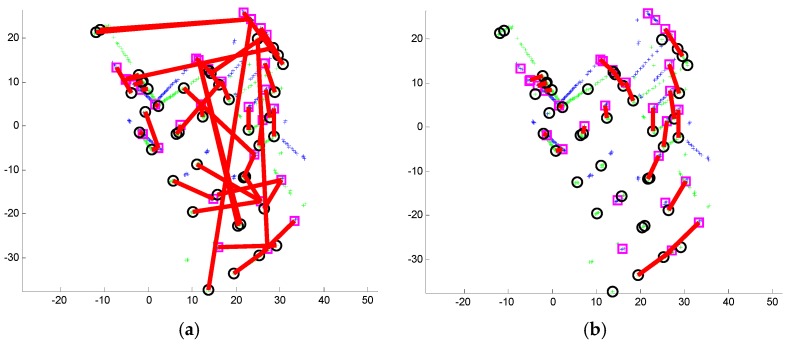
Feature point matching. (**a**) Point correspondences with outliers; (**b**) point matching result after outlier removal.

**Keypoint matching:** Suppose that we have detected the feature points *P_k_*_-1_ and *P_k_* of *S_k_*_-1_ and *S_k_*, respectively. As above, for each feature point *fp*_(*k,j*)_ ∊ *P_k_*_-1_, we compute the matching score between *fp*_(*k,j*)_ and all points in *P_k_*:

(5) $$score=∥dp(fp_{\left(k-1,i\right)})-dp(fp_{\left(k,j\right)})∥\;s.t.\;fp_{\left(k,j\right)}\in P_{k}$$

where dp(·) stands for the feature point descriptor. The feature point *fp*_(*k,j*)_ ∊ *P_k_* that achieves the best matching score will be accepted as the potential correspondence of *fp*_(*k,i*)_. As seen in Figure 5a, the points connected by a red line are potential correspondences. There are many false matches in the naive matching results. As we have obtained the rotation ${\bar{r}}_{k-1}$ between laser scans *S_k_*_-1_ and *S_k_* in the last section, we can eliminate the rotation changes between these potential correspondences. Thus, there is only translation differences between these transformed matching points. In fact, the correct point correspondences should have almost the same translations. We cluster the translations in *x* and *y* coordinates. The average translation ${\bar{t}}_{k-1}$ of the largest cluster in *x* and *y* is considered as the true translation between *S_k_*_-1_ and *Sk*. The correspondences that are inconsistent with the translation ${\bar{t}}_{k-1}$ are then rejected as outliers. The cleaned matching points are shown in Figure 5b.

### 3.4. Transformation Estimation

Let $C_{\left(k-1,k\right)}\{(fp_{\left(k-1,i\right)}\in P_{k-1},fl_{\left(k,i\right)}\in P_{k})\}$ be the feature point correspondence pairs; the relationship between pair $\left(fp_{\left(k-1,i\right)}\in P_{k-1},fl_{\left(k,i\right)}\in P_{k}\right)$ can by modeled by a rigid transformation:

(6) $$fp_{\left(k-1,i\right)}=r_{k-1}\cdot fl_{\left(k,i\right)}+t_{k-1}$$

The number of degrees of freedom of this equation is three, i.e., one for rotation and two for translation. Two correspondence pairs are enough to solve it. Classical approaches usually minimize the following nonlinear least-squares cost function via the Gauss-Newton [24] method:

(7) $$\underset{r_{k-1},t_{k-1}}{\arg\min}\sum_{i=1}^{n}{\left\|p_{i}-{\hat{p}}_{i}\right\|}_{2}^{2}$$

where $p_{i}=fp_{\left(k-1,i\right)}$ represents the observations, and ${\hat{p}}_{i}=r_{k-1}\cdot fl_{\left(k,i\right)}+t_{k-1}$ is the calculated value. $p_{i}$ and ${\hat{p}}_{i}$ are introduced only for compactness of notation. n is the number of pairs in $C_{\left(k-1,k\right)}$.

However, these methods are sensitive to outliers. They usually fail to obtain the correct solutions when the observations (feature correspondences) are corrupted by outliers. The current solutions usually combine minimal solvers with RANSAC-based [9] schemes to reject outliers during a prepro­cessing stage. Unfortunately, the minimal solvers with RANSAC-based schemes are sensitive to noise. In addition, RANSAC-based methods are very slow.

Outliers cannot be absolutely avoided in correspondences $C_{\left(k-1,k\right)}$, so a good solver should have the ability to automatically segment the residual vector $v=[v_{1},v_{2},...,v_{n}]$ into an inlier set $I(v_{i}|\left|v_{i}\right|\approx 0)$ and an outlier set $O(v_{i}|\left|v_{i}\right|\gg 0)$. Classical least-squares cost is based on a fundamental assumption that observations are subjected to a normal distribution and free of outliers. The results will be skewed. Recently, a sparsity-inducing norm, the lq-norm [25,26,27,28], has shown great potential and can achieve better performance than the l1-norm [29] and l0-norm [30]. Motivated by that, we reformulate the cost function based on the lq-norm metric:

(8) $$\underset{r_{k-1},t_{k-1}}{\arg\min}\sum_{i=1}^{n}{\left\|p_{i}-{\hat{p}}_{i}\right\|}_{q}^{q}$$

where ${\left\|\cdot \right\|}_{q}$ is an lq-norm (0<q<1) operator.

To optimize this problem, it is formed as an lq-norm penalized least-squares (lqLS) problem, which has been well studied by Marjanovic and Solo [28]. By introducing auxiliary variables $M=[m_{1},m_{2},...,m_{n}]$ into Equation (8), the cost function is rewritten as:

(9) $$\underset{r_{k-1},t_{k-1},M}{\arg\min}\sum_{i=1}^{n}{\left\|m_{i}\right\|}_{q}^{q}\;\mathrm{subject}\;\mathrm{to}\;\varepsilon_{i}=p_{i}-{\hat{p}}_{i}-m_{i}=0$$

Using the augmented Lagrangian function, we can reformulate this constrained optimization function into an unconstrained one:

(10) $$L_{\rho }(R,t,M,\Lambda )=\sum_{i=1}^{n}(∥m_{i}{∥}_{q}^{q}+\lambda_{i}^{T}\varepsilon_{i}+\frac{\rho }{2}∥\varepsilon_{i}{∥}_{2}^{2})=\sum_{i=1}^{n}(∥m_{i}{∥}_{q}^{q}+\frac{\rho }{2}∥\frac{\lambda_{i}}{\rho }+\varepsilon_{i}{∥}_{2}^{2}-\frac{1}{2\rho }∥\lambda_{i}{∥}_{2}^{2})$$

where $\Lambda =[\lambda_{1},\lambda_{2},...,\lambda_{n}]$ are Lagrange multipliers or dual variables and ρ>0 is a penalty parameter. 

To simplify this problem, the alternating direction method of multipliers (ADMM) is employed to decompose the function into three subproblems:

(11) $$\mathrm{prob}\;1:M^{m+1}:=\underset{M}{\arg\min}L_{\rho }(r_{k-1}^{m},t_{k-1}^{m},M,\Lambda^{m})==\underset{M}{\arg\min}\sum_{i=1}^{n}(∥m_{i}{∥}_{q}^{q}+\frac{\rho }{2}∥\delta_{i}-m_{i}{∥}_{2}^{2})$$

(12) $$\mathrm{prob}\;2:(r_{k-1}^{m+1},t_{k-1}^{m+1}):=\underset{r_{k-1},t_{k-1}}{\arg\min}L_{\rho }(r_{k-1},t_{k-1},M^{m},\Lambda^{m})=\underset{r_{k-1},t_{k-1}}{\arg\min}\sum_{i=1}^{n}(\frac{\rho }{2}∥\gamma_{i}-{\hat{p}}_{i}{∥}_{2}^{2})$$

(13) $$\mathrm{prob}\;3:\lambda_{i}^{m+1}:=\lambda_{i}^{m}+\rho \varepsilon_{i}\;i=1,2,...,n$$

where the superscript *m* denotes the iteration counter. $d_{i}=\frac{l_{i}}{\rho }+p_{i}+{\hat{p}}_{i}$ and $g_{i}=\frac{l_{i}}{\rho }+p_{i}-m_{i}$ are used only for compactness of notation. In **prob 1**, ***M*** is the only variable to be estimated, whereas the others are fixed. In **prob 2**, the rigid transformation ***T****_k_*_-1_ = {***r****_k_*_-1_,***t****_k_*_-1_} is the only variable. The ADMM alternates among these three steps until convergence. Details about augmented Lagrangian methods and ADMM can be found in the literature [31].

**prob 1** is an lq-norm penalized least squares (lqLS) problem. We adapt Marjanovic and Solo’s lq Cyclic Descent (lqCD) algorithm [28] to solve it. More details about the lqLS problem may be seen in Marjanovic and Solo’s literature [28]. **prob 2** is a classical least-squares function, which can be easily solved by DLT. Our lq-norm solver can converge to the correct solution within only a few iterations because the very good initial guess has been obtained above.

## 4. Results

### 4.1. SLAM System and Datasets

**Figure 6:**
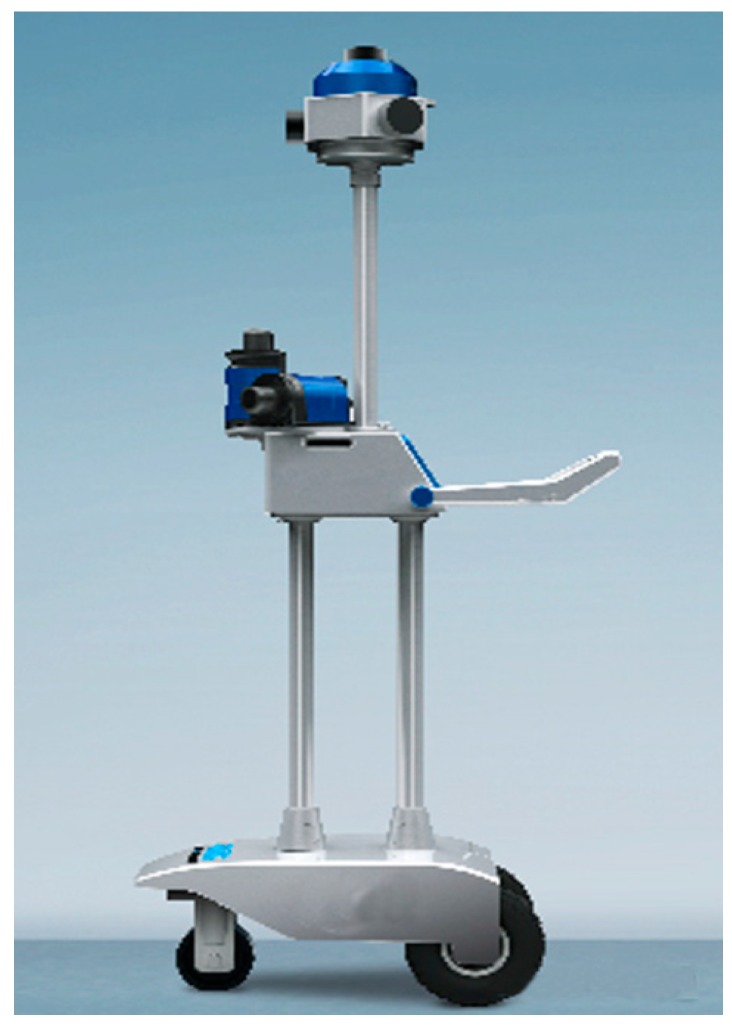
Our SLAM system. It consists of three laser scanners, a panoramic camera, and two odometers.

We design a hardware system for indoor mapping and modeling tasks. As shown in Figure 6, this SLAM system consists of three laser scanners, a panoramic camera, and two odometers. The 2D Sick laser scanner has a 360° field of view, a 0.5°-point measurement resolution, and a 10 Hz scanning rate. The horizontal laser is used for location (scan matching), and the other two oblique lasers are used for mapping 3D point clouds. The two odometers can provide a good initial guess for ICP scan matching.

We collect two datasets using this hardware system. The first one is a market with an area of 8000 m^2^. We push the cart and walk. The length of the full trajectory is 1137 m. We sample this dataset into 2200 key laser scans. The second one is an underground parking garage with an area of 3200 m^2^. The length of the trajectory is 357 m. It is sampled in 1414 key laser scans. In both datasets, the moving speed of the cart is 1 m/s.

Moreover, we also use two SLAM benchmarking datasets for evaluation [32]. The first dataset was collected at the Intel Research Lab, and the other one was collected at the MIT CSAIL Building. The ground truth of both datasets was provided.

### 4.2. Comparison with State-of-the-Art Methods

For our datasets, we randomly pick 4 laser scan pairs from each dataset and register these scan pairs by using PSM, PLICP, ICP without an initial guess (initial guess set to zero, denoted by ICP^1^), the proposed method and ICP with a good initial guess (initial-guess obtained by the two odometers, denoted by ICP^2^). The results of ICP^2^ are regarded as the ground truth. For benchmarking datasets, we randomly pick 50 laser scan pairs. We compare our method with PSM, PLICP and ICP^1^. The ICP^1^ uses a point-to-point metric and sets the maximum iterations to 100. The source codes of PSM and PLICP are obtained from the authors’ websites. The source code of ICP used in this paper can be found in [33].

**Figure 7:**
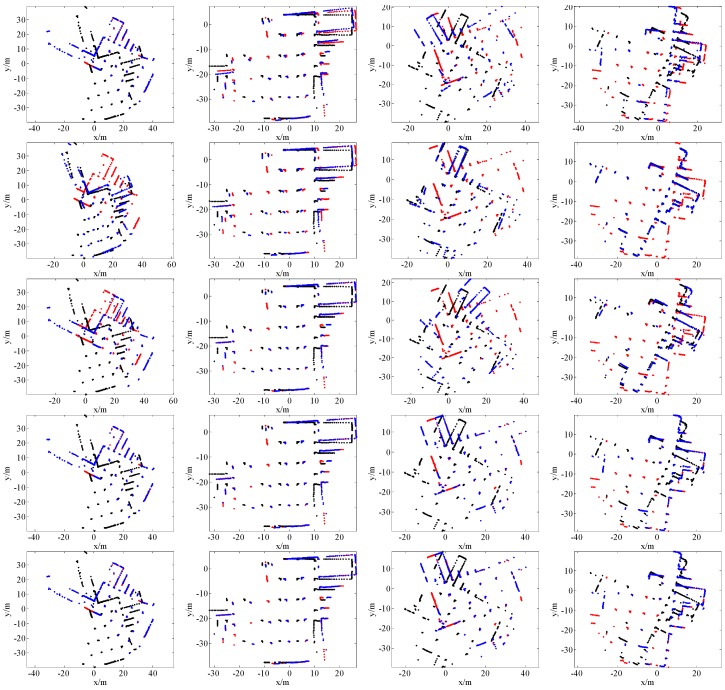
Some scan matching results of dataset 1. The first row is the results of PSM; the second row is the results of PLICP; the third row is the results of ICP without an initial guess (ICP^1^); the fourth row is our scan matching results; the last row is the results of ICP with a good initial guess (ICP^2^). In all of these plots, the points denoted by red +, black +, and blue + represent the first laser scan *S_k_*_-1_, the second laser scan *S_k_*, and the transformed laser scan of *S_k_* by using the estimated transformation matrix, respectively.

**Figure 8:**
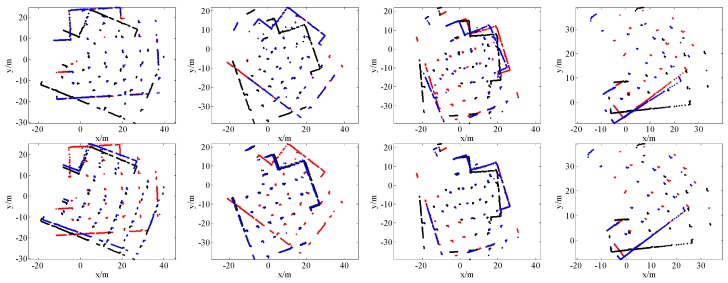

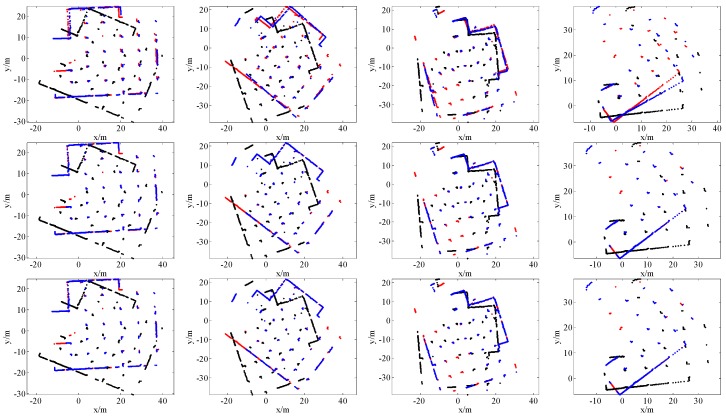
Some scan matching results of dataset 2. The first row is the results of PSM; the second row is the results of PLICP; the third row is the results of ICP without an initial guess (ICP^1^); the fourth row is our scan matching results; the last row is the results of ICP with a good initial guess (ICP^2^). In all of these plots, the points denoted by red +, black +, and blue + represent the first laser scan *S_k_*_-1_, the second laser scan *S_k_*, and the transformed laser scan of *S_k_* by using the estimated transformation matrix, respectively.

**Figure 9:**
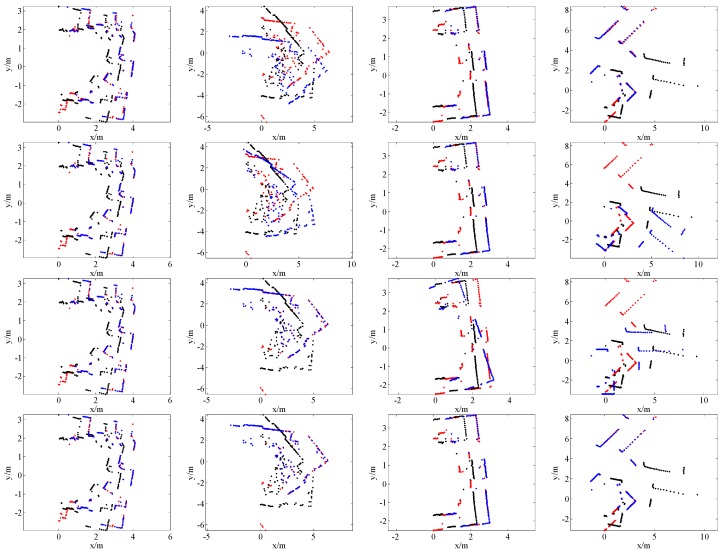

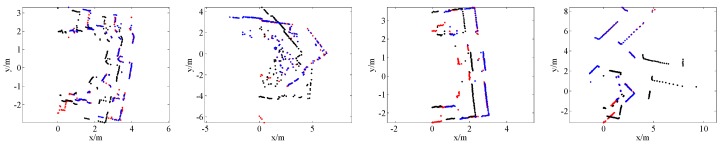
Some scan matching results of the Intel dataset. The first row is the results of PSM; the second row is the results of PLICP; the third row is the results of ICP without an initial guess (ICP^1^); the fourth row is our scan matching results; the last row is the ground truth. In all these plots, the points denoted by red +, black +, and blue + represent the first laser scan *S_k_*_-1_, the second laser scan *S_k_*, and the transformed laser scan of *S_k_* by using the estimated transformation matrix, respectively.

**Figure 10:**
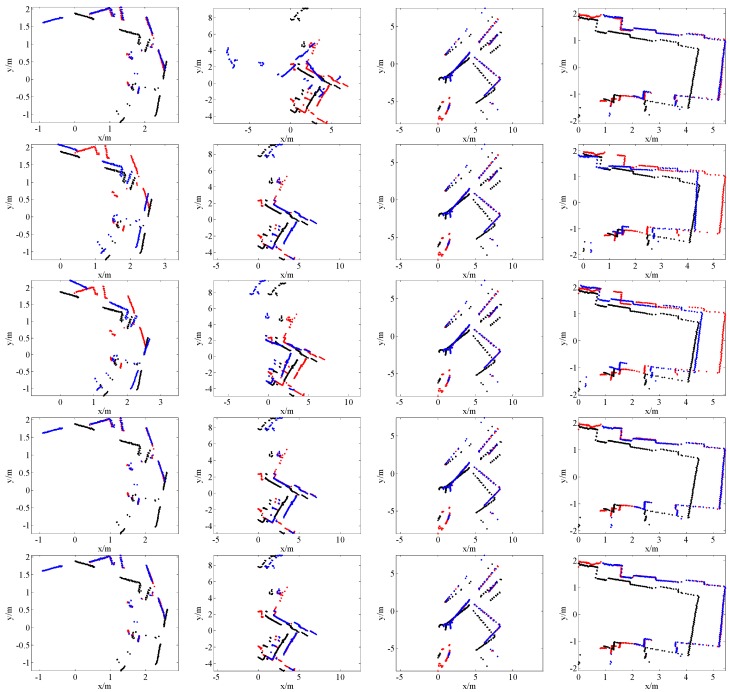
Some scan matching results of the MIT dataset. The first row is the results of PSM; the second row is the results of PLICP; the third row is the results of ICP without an initial guess (ICP^1^); the fourth row is our scan matching results; the last row is the ground truth. In all these plots, the points denoted by red +, black +, and blue + represent the first laser scan *S_k_*_-1_, the second laser scan *S_k_*, and the transformed laser scan of *S_k_* by using the estimated transformation matrix, respectively.

Figure 7, Figure 8, Figure 9 and Figure 10 give the visual matching results. As seen, our method achieves similar results to ICP^2^ or the ground truth. In all plots of our results, ICP^2^ or the ground truth, the transformed laser scan of *S_k_* (denoted by blue +) covers the first laser scan *S_k_*_-1_ (denoted by red +). This means that both our method and ICP^2^ can correctly align these laser scan pairs. PLICP and ICP^1^ have similar performances; PSM performs better than PLICP and ICP^1^. However, PSM, PLICP and ICP^1^ will fail in some cases. These methods are very sensitive to the initial guess, whereas our method is initial free.

**Figure 11:**
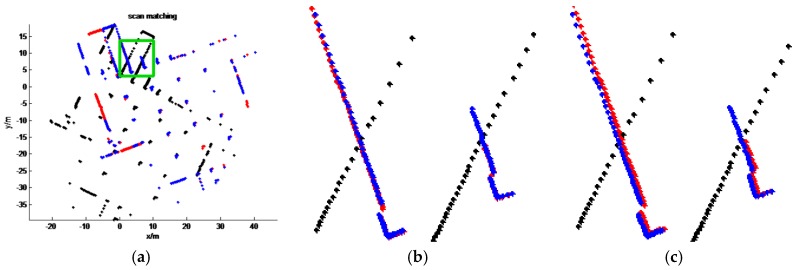
The enlarged result of scan pair 3 of dataset 1. (**a**) The green box is the enlarged region; (**b**) the enlarged results of our method; (**c**) the enlarged results of ICP^2^.

Table 1 and Table 2 show the quantitative evaluation results of our datasets. In the Tables, *tx* and *ty* represent the relative translation along the *x*-axis and *y*-axis between a laser scan pair, respectively; rθ represents the relative rotation angle between a scan pair. In this evaluation, we regard the results of ICP^2^ as the approximate ground truth rigid transformation. PSM, PLICP, ICP^1^ and our method are compared with ICP^2^. From these tables, we can also draw similar conclusions as in the visual comparisons. As seen, our estimated transformations are almost the same as ICP^2^. The maximum angle difference between our method and ICP^2^ is less than 0.01 rad (0.573°). For most cases, the translation differences between our method and ICP^2^ are smaller than 0.05 m. The maximum translation difference is 0.09 m, which is the result of scan pair 3 in dataset 1. We enlarge the visual results of scan pair 3 (see Figure 11) and find that our result is even better than that of ICP^2^. The success rates of PSM, PLICP and ICP^1^ are 37.5%, 12.5% and 12.5%, respectively. Comparing ICP^1^ with ICP^2^, only the second scan pair is correctly matched; the translation differences are 0.07 m and 0.04 m, which are less accurate than our method. Even the translation differences of the third scan pair in dataset 2 are 0.13 m and 0.13 m, and their rotation difference is up to 0.05 rad (2.865°). ICP^1^ can succeed only for scan pair 2 of dataset 1, which can be expected because the rotation between this pair is small. Thus, the proposed method is much more reliable when the initial guess is unavailable.

Table 3 gives the quantitative evaluation results of the benchmarking datasets. In this table *e_x_*, *e_y_* and *e*_θ_ represent the mean absolute error of translation and rotation. Their calculation equations are as follows:

(14) $$e_{x}=\frac{\sum_{i=1}^{n}\left|tx-tx_{gt}\right|}{n},\;e_{y}=\frac{\sum_{i=1}^{n}\left|ty-ty_{gt}\right|}{n},\;e_{\theta }=\frac{\sum_{i=1}^{n}\left|t\theta -t\theta_{gt}\right|}{n}$$

where *tx_gt_* and *ty_gt_* are the ground truth of translation; *t*θ*_gt_* is the ground truth of rotation; and *n* is the total scan pairs (in this experiment, *n* = 50). If *e_x_* and *e_y_* of a scan pair are smaller than 0.1 m and *e*_θ_ is smaller than 0.03 rad, this estimated transformation matrix is regarded as a correct one. The success rate is the ratio of correctly matched scan pairs and total scan pairs. From this table, we can see that the translation error of our method is less than 2 cm and that the rotation error is approximately 0.1 rad. This is much better than those of PSM, PLICP and ICP^1^ because the success rate of our method is 100%. Our method achieves 18%, 34% and 42% growth rates of the success rate for the Intel dataset and 20%, 50% and 56% growth rates for the MIT dataset, respectively. PSM, PLICP and ICP^1^ are not very robust and stable because of the need for an initial guess.

### 4.3. Running Time

Scan matching is usually a front end of SLAM systems, in which time-consuming methods are unacceptable. We select four scan pairs for evaluation. The numbers of points in each scan of the four scan pairs are 180, 360, 720 and 1080. All experiments are run in Microsoft Visual Studio 2010 installed on a laptop PC with an Intel Core i5-3210M 2.5 GHz CPU and 8 GB of RAM. Note that we rewrite the ICP code in C++ for comparison. The results are given in Table 4.

As seen, PSM is the fastest. It achieves 100 FPS when the number of points is 720. Our method is much faster than PLICP and ICP^1^ because the numbers of keypoints and keylines are very small. Our method can achieve almost 30 FPS and 20 FPS when the numbers of points are 360 and 720, respectively. This is sufficient for 2D indoor scan matching because the number of points in a 2D laser scan is less than 1080 in most cases.

### 4.4. Application for SLAM 

For each dataset, we first perform the proposed scan matching algorithm on all consecutive key laser scan pairs, obtaining the relative positions and orientations ***T****_k_*_-1_ = {***r****_k_*_-1_,***t****_k_*_-1_} of these pairs. We choose the first key laser scan as the base on which to build a global map. The coordinate system of this global map is the same as the first key scan. We use *Q_k_*_-1_ to represent the points on the incremental global map, accumulated until the *k-1*-th laser scan. For a new laser scan *S_k_*, we then match *S_k_* with *Q_k_*_-1_ via ICP. The initial guess of ICP is provided by our method (***T****_k_*_-1_ = {***r****_k_*_-1_,***t****_k_*_-1_})). This transformation matrix ***T****_k_*_-1_ = {***r****_k_*_-1_,***t****_k_*_-1_} is refined by this process, and more accurate relative positions and orientations are achieved. The global map is also updated by fusing the points of laser scan *S_k_*. This map matching technique has a very important advantage: it is a global matching method, which can largely reduce the drift caused by scan matching methods. After all laser scans are processed, a 2D indoor map and a trajectory can be produced. Finally, this trajectory (positions and orientations) can be further optimized by some global optimization approaches such as G2O [34] or some filtering techniques. In this experiment, we regard the trajectory refined by the global map as the initial guess needed by particle filter SLAM (Gmapping [35]) and obtain a more accurate indoor map. We can also generate the 3D point clouds of the indoor scenes based on the other two laser scanners and the optimized trajectory.

**Figure 12:**
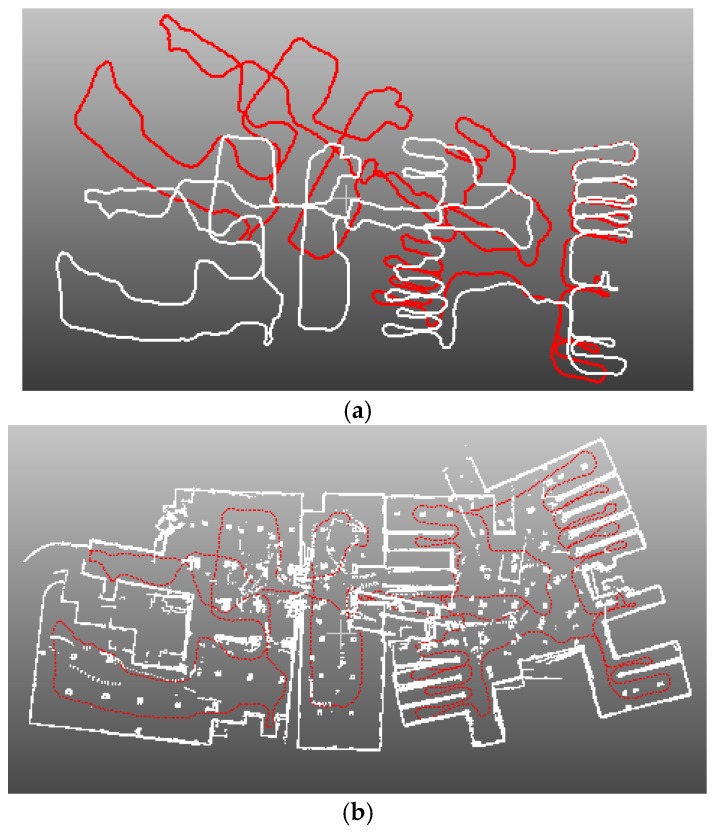

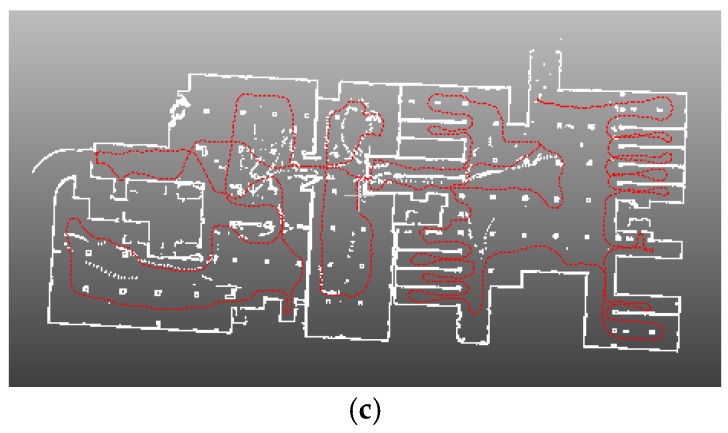
(**a**) trajectory comparison. Red line is the trajectory of our method before Gmapping refinement; white line is the result after refinement; (**b**) 2D map built by trajectory without refinement; (**c**) 2D map built by trajectory with Gmapping refinement.

**Figure 13:**
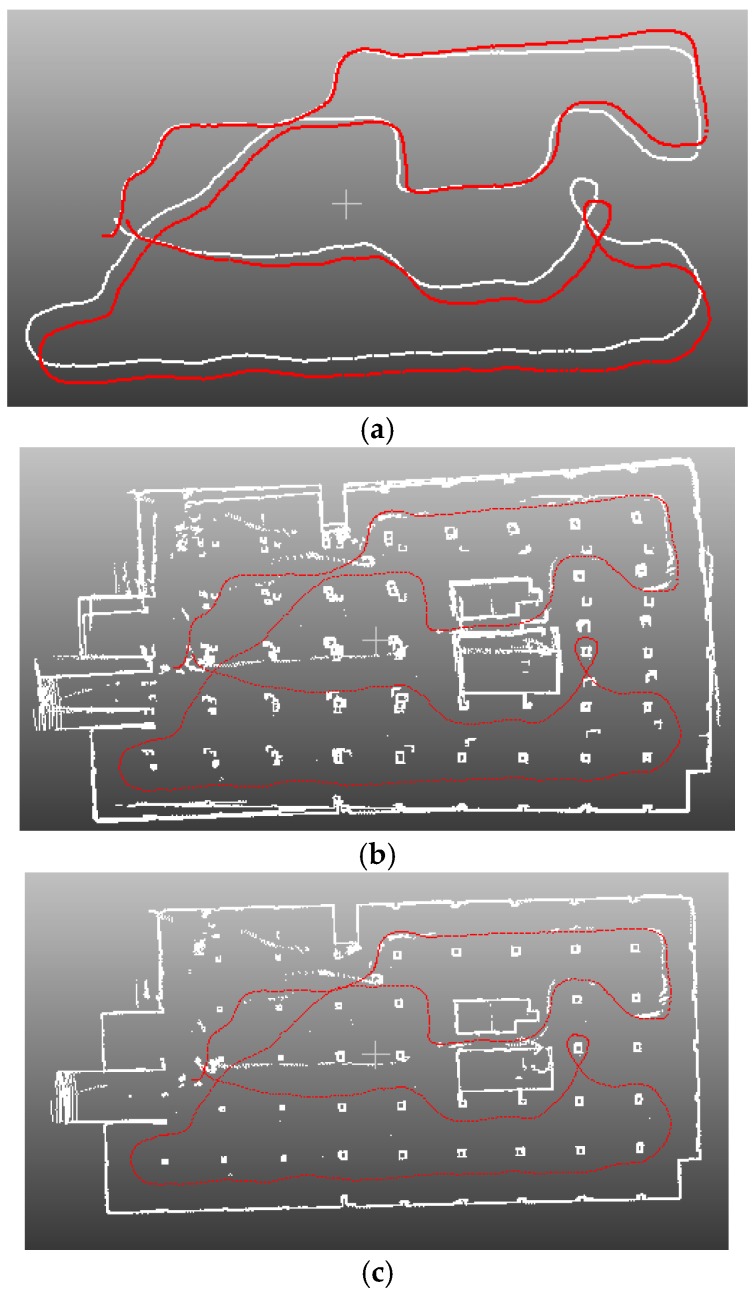
(**a**) trajectory comparison. Red line is the trajectory of our method before Gmapping refinement; white line is the result after refinement; (**b**) 2D map built by trajectory without refinement; (**c**) 2D map built by trajectory with Gmapping refinement.

**Figure 14:**
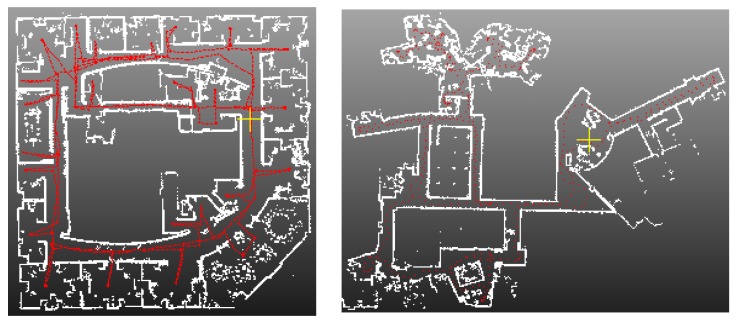
The final results of Intel and MIT datasets. (**Left**) Intel; (**right**) MIT.

**Figure 15:**
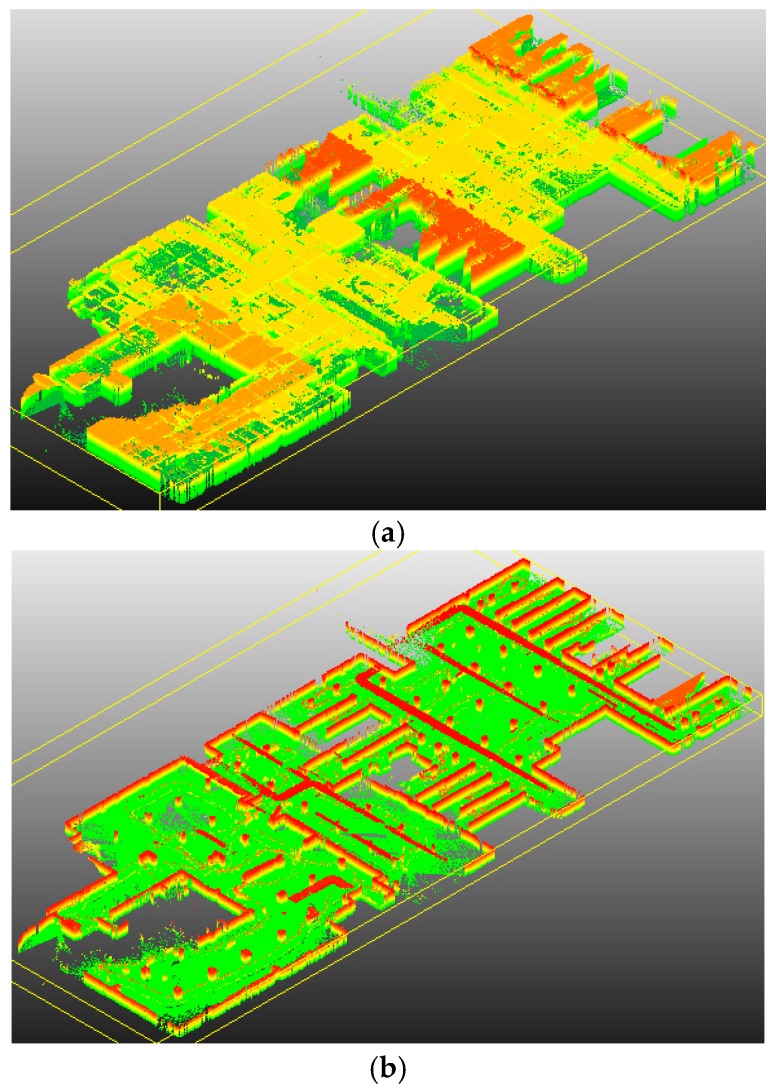
The 3D point clouds of dataset 1. (**a**) 3D point clouds; (**b**) Point clouds after cutting off the roof.

Figure 12 and Figure 13 show the indoor mapping results of dataset 1 and dataset 2, respectively. As seen, a smooth trajectory can be achieved by combining our scan matching approach with a map matching technique. There are no abrupt changes in the results, which indicates that there are no obvious false matches because the cart is moved at a near-constant speed. The unrefined trajectory has a similar shape to the global refined one. Its local accuracy is much closer to the global trajectory. The proposed method drifts over time, which could not be avoided by any scan matching method. From the second pictures in Figure 12 and Figure 13, we can see that the 2D point clouds have very high local accuracy. The thickness of the point clouds is less than 0.1 m in most cases. The point clouds represent the outline of indoor walls whose thickness is almost 0 in the ideal case. However, the range noise of the Sick laser scanner is 0.02 m, and the location error cannot be avoided. Thus, a trajectory that can construct a map with 0.1 m local accuracy is sufficient to provide a good initial guess for global refinement methods, such as Gmapping. The third pictures of Figure 12 and Figure 13 verify this conclusion. The thickness of the point clouds is less than 0.08 m in all cases. There is no “ghosting” in the maps, and the walls are straight. The constructed 2D maps are consistent with the real scenes. Figure 14 gives the final indoor maps of the Intel and MIT datasets. Figure 15 shows the 3D point clouds of dataset 1 reconstructed by the other two oblique lasers.

### 4.5. Limitations

There are still some problems in our algorithm that must be resolved. First, our method is unsuitable for outdoor scenes. Outdoor scenes are more complicated than indoor environments. There are many moving objects and few line segments in outdoor scenes that will affect our method. This is an inherent drawback of our scan matching. Second, our scan matching method has only three degrees of freedom. It will fail if the floor of the indoor scene is not planar. Thus, if we want to build a map of a multiple-floor indoor environment, we must perform the process for each floor; all floors are then registered manually. This is a common drawback of 2D scan matching methods. In addition, our method highly relies on the feature detection stage. Our method needs at least one correct line correspondence and two correct point correspondences. If there are no line correspondences in a laser scan pair, we should discard one scan and obtain another one to build a new pair. This is the process of key laser scan selection. Alternatively, we can use only point correspondence for scan matching. If there are few 1D SIFT feature points, we should add other feature points, such as normal-based feature points, curve-based feature points, line intersection points, and so on.

## 5. Conclusions

In this paper, we propose a 2D laser scan matching method based on point and line features. Our method does not require an initial guess for the transformation. We carefully design a framework for the detection of point and line feature correspondences. The feature points are detected based on an extended 1D SIFT, and line features are extracted via a modified split-and-merge algorithm. Line segments are a very important type of features for indoor environments. The advantages of using line features are twofold: first, line features are more distinct for indoor scenes and are less sensitive to noise in the laser range measurements; second, the orientation information can be easily extracted from 2D line correspondences, which can help us reject feature point outliers and provide a good initial guess for our transformation estimation stage. The point and line features are then described by a distance histogram. The histogram cluster technique can help us filter outliers and provide an accurate initial value of the rigid transformation. We also propose a new relative pose estimation method that is robust to outliers. Unlike the conventional RANSAC-based strategy, there is no gross error detection stage in our pose estimation algorithm. In addition, our pose estimation algorithm is more robust to noise than the RANSAC-based one. Extensive experiments on real data demonstrate that the proposed method is almost as accurate as ICPs and is initial free. We also show that our scan matching method can be integrated into a simultaneous localization and mapping (SLAM) system for indoor mapping. There are also some limitations to our method. The main limitation is the three degrees of freedom. Our future work will be to extend the proposed method to six degrees of freedom.

## Figures and Tables

**Table 1:** Quantitative evaluation on dataset 1.

Data	Scan Pair 1			Scan Pair 2			Scan Pair 3			Scan Pair 4		
Methods	*tx*/m	*ty*/m	*r*θ/rad	*tx*/m	*ty*/m	*r*θ/rad	*tx*/m	*ty*/m	*r*θ/rad	*tx*/m	*ty*/m	*r*θ/rad
PSM	1.22	0.55	0.84	0.51	0.09	0.11	−1	−0.62	1	−0.29	0.57	0.33
PLICP	1.03	0.61	0.13	0.16	0	0.07	0.56	-0.08	0.08	0.01	−0.25	0
ICP^1^	6.94	−2.76	0.82	1.46	0.13	0.07	7.63	3.76	−0.3	1.84	−0.27	−0.05
ours	1.21	0.58	0.83	1.55	0.05	0.07	1.9	0.64	0.79	2.08	0.31	0.3
ICP^2^	1.24	0.55	0.84	1.53	0.09	0.07	1.85	0.55	0.8	2.07	0.31	0.3
*d*(PSM–ICP^2^)	0	0	0	−1.06	0	0.04	−2.87	−1.28	0.2	−2.41	0.24	0.02
*d*(PLICP–ICP^2^)	−0.21	0.06	−0.71	−1.37	−0.09	0	−1.29	−0.63	−0.72	−2.06	−0.56	−0.3
*d*(ICP^1^–ICP^2^)	5.7	−3.31	−0.02	−0.07	0.04	0	5.78	3.21	−1.1	−0.23	−0.58	−0.35
*d*(ours–ICP^2^)	−0.03	0.03	−0.01	0.02	−0.04	0	0.05	0.09	−0.01	0.01	0	0

**Table 2:** Quantitative evaluation on dataset 2.

Data	Scan Pair 1			Scan Pair 2			Scan Pair 3			Scan Pair 4		
Methods	*tx*/m	*ty*/m	*r*θ/rad	*tx*/m	*ty*/m	*r*θ/rad	*tx*/m	*ty*/m	*r*θ/rad	*tx*/m	*ty*/m	*r*θ/rad
PSM	0.68	0.1	0.45	1.34	0.55	0.59	−0.56	−0.78	0.31	−1.36	−1.91	0.46
PLICP	0.5	1.46	0.04	−0.62	−0.05	0.02	1.85	0.22	0.21	0.42	−0.62	0.54
ICP^1^	1.19	0.12	0.43	2.45	1.07	0.52	1.98	0.34	0.17	1.87	0.51	0.4
ours	0.65	0.07	0.45	1.34	0.54	0.58	1.85	0.25	0.22	1.94	0.55	0.54
ICP^2^	0.64	0.08	0.44	1.37	0.55	0.58	1.85	0.21	0.22	1.96	0.55	0.54
*d*(PSM–ICP^2^)	0.04	0.02	0.01	−0.03	0	0.01	−2.41	−0.99	0.09	−3.32	−2.46	−0.08
*d*(PLICP–ICP^2^)	−0.14	1.38	−0.4	−1.99	−0.6	−0.56	0	0.01	−0.01	−1.54	−1.17	0
*d*(ICP^1^–ICP^2^)	0.55	0.04	−0.01	1.08	0.52	−0.06	0.13	0.13	−0.05	−0.09	−0.04	−0.14
*d*(ours–ICP^2^)	0.01	−0.01	0.01	−0.03	−0.01	0	0	0.04	0	−0.02	0	0

**Table 3:** Quantitative evaluation on Intel and MIT datasets.

Datasets	Intel				MIT			
Method	*e_x_*/m	*e_y_*/m	*e*_θ_/rad	Success Rate	*e_x_*/m	*e_y_*/m	*e*_θ_/rad	Success Rate
PSM	0.06	0.22	0.08	82%	0.28	0.09	0.13	80%
PLICP	0.17	0.22	0.39	66%	0.53	0.29	0.26	50%
ICP^1^	0.44	0.36	0.41	58%	0.58	0.27	0.25	44%
Ours	0.02	0.01	0.01	100%	0.01	0.02	0.01	100%

**Table 4:** The results of computation time.

Methods	180/ms	360/ms	720/ms	1080/ms
PSM	2	5	10	14
PLICP	32	96	252	364
ICP^1^	37	117	286	372
Ours	15	34	57	92
